# ﻿Two new species of *Neohelicomyces* (Tubeufiaceae, Tubeufiales) from Hainan Province, China

**DOI:** 10.3897/mycokeys.126.174186

**Published:** 2025-12-04

**Authors:** Fan Gao, Ting-Hong Tan, Song Bai, Chun-Fang Wu, Ning-Ning Zhao, Na Qiu, Min Zhou, Jian Ma

**Affiliations:** 1 School of Agriculture and Forestry Engineering and Planning, Tongren University, Tongren, Guizhou 554300, China; 2 Guizhou Provincial Key Laboratory for Biodiversity Conservation and Utilization in the Fanjing Mountain Region, Tongren University, Tongren, Guizhou 554300, China; 3 Guizhou Industry Polytechnic College, Guiyang, Guizhou 550008, China; 4 School of Food and Pharmaceutical Engineering, Guizhou Institute of Technology, Guiyang, Guizhou 550003, China

**Keywords:** Dothideomycetes, helicosporous hyphomycetes, phylogeny, taxonomy, two new species

## Abstract

During a survey of helicosporous hyphomycetes in tropical regions, four fungal strains were isolated from decaying wood in terrestrial habitats of Hainan Province, China. Based on combined phylogenetic analyses of LSU, ITS, *tef*1-*α*, and *rpb2* sequence data, together with morphological evidence, two novel species, *Neohelicomyces terrestris* and *N. tropicus*, are herein proposed. Comprehensive descriptions, illustrations, taxonomic notes, and phylogenetic analyses are provided to confirm the taxonomic placement. The newly described species were found in tropical rainforests, thereby expanding the known distribution of *Neohelicomyces* in tropical terrestrial habitats.

## ﻿Introduction

Helicosporous hyphomycetes are a group of asexual fungi characterized by helicoid or spiral conidia (Lu and Kang 2020; Ma et al. 2024b). To date, reported helicosporous hyphomycetes belong to three phyla (Ascomycota, Basidiomycota, and Zoopagomycota), 10 classes (Agaricomycetes, Atractiellomycetes, Dothideomycetes, Eurotiomycetes, Exobasidiomycetes, Leotiomycetes, Orbiliomycetes, Sordariomycetes, Tremellomycetes, and Zoopagomycetes), 20 orders (Agaricales, Atractiellales, Chaetosphaeriales, Exobasidiales, Helotiales, Hypocreales, Lulworthiales, Microascales, Microthyriales, Mycosphaerellales, Mytilinidiales, Orbiliales, Pleosporales, Pleurotheciales, Sclerococcales, Torpedosporales, Tremellales, Tubeufiales, Venturiales, and Zoopagales), 25 families, 103 genera, and nine fossil genera (Morgan 1892; Moore 1957; Rao and Rao 1964; Goos 1985; Tsui et al. 2006; Boonmee et al. 2011, 2014, 2021; Hyde et al. 2016; Singh and Singh 2016; Chaiwan et al. 2017; Dai et al. 2017; Doilom et al. 2017; Tibpromma et al. 2018; Li et al. 2022; Tian et al. 2022). Among them, the Tubeufiaceae (Tubeufiales, Dothideomycetes) is the most species-rich and morphologically diverse group of helicosporous hyphomycetes (Lu et al. 2017, 2018a, 2018b, 2022, 2023b; Luo et al. 2017; Ma et al. 2023, 2024b, 2025). Helicosporous genera within Tubeufiaceae, such as *Helicoma*, *Helicomyces*, *Helicosporium*, *Neohelicomyces*, *Neohelicosporium*, and *Tubeufia*, represent important fungal resources capable of producing secondary metabolites with novel chemical structures and notable bioactivities (Lu et al. 2018b; Lu and Kang 2020; Zeng et al. 2022; Qian et al. 2023; Zhang et al. 2023a, 2023b, 2024; Zheng et al. 2023). These compounds show considerable potential in drug development, including antitumor, anticancer, and antibacterial applications (Ma et al. 2024b).

*Neohelicomyces* Z.L. Luo, D.J. Bhat & K.D. Hyde was established by Luo et al. (2017) with *N. aquaticus* as the type species, based on phylogenetic analyses of combined LSU, ITS, and *tef1-α* sequence data and morphological characteristics. Currently, *Neohelicomyces* comprises 32 accepted species (Luo et al. 2017; Lu et al. 2018b, 2022; Tibpromma et al. 2018; Crous et al. 2019a, 2019b; Dong et al. 2020; Hsieh et al. 2021; Yang et al. 2023; Ma et al. 2024b; Lin et al. 2025; Peng et al. 2025; Sun et al. 2025). Species of *Neohelicomyces* are distributed across China, the Czech Republic, Germany, Italy, Japan, the Netherlands, Thailand, and the USA, occurring as saprobes on various substrates such as bamboo culms, *Deschampsia cespitosa*, *Fraxinus excelsior*, *Melaleuca styphelioides*, *Miscanthus floridulus*, *Pandanus* sp., *Quercus robur*, and decaying wood in both freshwater and terrestrial habitats (Linder 1929; Goos 1986, 1989, 1990; Tsui et al. 2006; Zhao et al. 2007; Ruibal et al. 2009; Luo et al. 2017; Lu et al. 2018b, 2022; Tibpromma et al. 2018; Crous et al. 2019a, 2019b; Dong et al. 2020; Hsieh et al. 2021; Yang et al. 2023; Ma et al. 2024b; Lin et al. 2025; Peng et al. 2025; Sun et al. 2025). The asexual morph of *Neohelicomyces* is characterized by gregarious, white, grayish-brown, yellowish-green, and pinkish colonies; macronematous, mononematous, erect, septate, pale brown, branched and/or unbranched conidiophores; mono- to polyblastic, integrated, terminal or intercalary conidiogenous cells with denticles; and acropleurogenous or pleurogenous, aseptate or septate, guttulate, hyaline, helicoid conidia (Yang et al. 2023; Ma et al. 2024a, 2024b; Peng et al. 2025). The sexual morph is characterized by superficial, solitary, scattered, reddish brown to brown, subglobose ascomata; eight-spored, bitunicate, cylindric-clavate, rounded-at-apex, short-pedicellate asci; and multiseriate, narrowly cylindrical, straight to slightly curved, hyaline to pale brown, septate, rough ascospores (Sun et al. 2025).

In this study, four isolates of helicosporous hyphomycetes, representing two distinct taxonomic lineages, were collected from terrestrial environments in Hainan Province, China. Comprehensive analyses, including detailed morphological observations, illustrations, and multigene phylogenetic assessments, were conducted to accurately characterize these isolates. Based on the integrative evidence, two previously undescribed species are proposed and formally introduced here: *Neohelicomyces terrestris* and *N. tropicus*. These findings not only expand the current understanding of the genus *Neohelicomyces* but also provide valuable insights into its diversity in tropical terrestrial habitats.

## ﻿Materials and methods

### ﻿Sample collection, specimen examination, and isolation

Decaying wood was collected from Hainan Province, southwestern China. Samples were taken to the laboratory in plastic bags with the collection details, including localities and dates (Rathnayaka et al. 2024). The microscopic features were examined and photographed using a stereomicroscope (SMZ-168, Nikon, Japan) and an ECLIPSE Ni compound microscope (Nikon, Tokyo, Japan) with a Canon 90D digital camera (Canon, China). Measurements were made using Tarosoft (R) Image Frame Work software. Photo plates were assembled using Adobe Photoshop CC 2019 (Adobe Systems, USA).

Single-spore isolation was performed following the methods described by Senanayake et al. (2020), and the germinated conidia were aseptically transferred to fresh PDA plates. Morphological characters of fungal colonies, including color, shape, and size, were documented. Dried specimens were deposited in the Herbarium of the Kunming Institute of Botany, Chinese Academy of Sciences (Herb. HKAS), Kunming, China, and the Herbarium of the Guizhou Academy of Agriculture Sciences (Herb. GZAAS), Guiyang, China. Pure cultures were deposited in the Guizhou Culture Collection (GZCC), Guiyang, China. MycoBank numbers of the newly obtained species were registered in the MycoBank database (https://www.mycobank.org/).

### ﻿DNA extraction, PCR amplification, and sequencing

Fresh fungal mycelia were scraped from colonies grown on PDA plates and transferred to a 1.5 mL microcentrifuge tube using a sterilized lancet for genomic DNA extraction. Genomic DNA was extracted using the Biospin Fungus Genomic DNA Extraction Kit (BioFlux, China). LR0R/LR5, ITS5/ITS4, EF1-983F/EF1-2218R, and fRPB2-5F/fRPB2-7cR were employed to amplify the large ribosomal subunit (LSU; Vilgalys and Hester 1990), internal transcribed spacer (ITS; White et al. 1990), translation elongation factor 1-alpha (*tef*1-*α*; Rehner and Buckley 2005), and RNA polymerase II second-largest subunit (*rpb2*; Liu et al. 1999) sequence fragments, respectively. DNA preparation was conducted in a 25 μL mixture, which included 1 μL DNA, 1 μL each of the forward and reverse primers, and 22 μL of 1.1× T3 Super PCR Mix (including 8.5 μL distilled-deionized water; Tsingke Biotech, Chongqing, China). The conditions for the polymerase chain reaction (PCR) correspond to those reported by Ma et al. (2023). The PCR products were purified and sequenced with the same primers at Beijing Tsingke Biotechnology Co., Ltd.

### ﻿Phylogenetic analyses

The newly obtained sequences were checked and assembled using BioEdit v.7.0.5.3 (Hall 1999) and SeqMan v.7.0.0 (DNASTAR, Madison, WI, USA; Swindell and Plasterer 1997), respectively. The sequences incorporated in this study were downloaded from GenBank (Table 1; https://www.ncbi.nlm.nih.gov/). Multiple sequences were aligned using MAFFT v.7.473 (https://mafft.cbrc.jp/alignment/server/; Katoh et al. 2019). The dataset was trimmed using trimAl v.1.2rev59 software (Capella-Gutiérrez et al. 2009). A combined sequence dataset was created using SequenceMatrix-Windows-1.7.8 software (Vaidya et al. 2011).

**Table 1.** Taxa used in this study and their GenBank accession numbers.

Taxon	Strain	GenBank Accessions			
		LSU	ITS	*tef*1-α	*rpb*2
*Helicotubeufia hydei*	MFLUCC 17-1980^T^	MH290026	MH290021	MH290031	MH290036
*Helicotubeufia jonesii*	MFLUCC 17-0043^T^	MH290025	MH290020	MH290030	MH290035
*Neohelicomyces acropleurogenus*	CGMCC 3.25549^T^	PP639450	PP626594	PP596351	PP596478
*Neohelicomyces aquaticus*	MFLUCC 16-0993^T^	KY320545	KY320528	KY320561	MH551066
*Neohelicomyces aquisubtropicus*	GZCC 23-0080^T^	PQ098537	PQ098499	PV768327	PV768336
*Neohelicomyces aseptatus*	CGMCC 3.25564^T^	PP639451	PP626595	PP596352	PP596479
*Neohelicomyces astrictus*	HKAS 105122^T^	PQ898796	PQ898760	PV040811	N/A
*Neohelicomyces brunneus*	HKAS 105147^T^	PQ898805	PQ898768	PV040818	N/A
*Neohelicomyces dehongensis*	MFLUCC 18-1029^T^	MN913709	NR_171880	MT954393	N/A
*Neohelicomyces denticulatus*	GZCC 19-0444^T^	MW133855	OP377832	N/A	N/A
*Neohelicomyces deschampsiae*	CPC 33686^T^	MK442538	MK442602	N/A	N/A
*Neohelicomyces edgeworthiae*	CGMCC 3.25565^T^	PP639453	PP626597	PP596354	PP596481
*Neohelicomyces grandisporus*	KUMCC 15-0470^T^	KX454174	KX454173	N/A	MH551067
*Neohelicomyces guizhouensis*	GZCC 23-0725^T^	PP512973	PP512969	PP526727	PP526733
*Neohelicomyces guttulatus*	CGMCC 3.25550^T^	PP639454	PP626598	PP596355	N/A
*Neohelicomyces hainanensis*	GZCC 22-2009^T^	OP508774	OP508734	OP698085	OP698074
*Neohelicomyces helicosporus*	GZCC 23-0633^T^	PP512975	PP512971	PP526729	PP526735
*Neohelicomyces hyalosporus*	GZCC 16-0086^T^	MH558870	MH558745	MH550936	MH551064
*Neohelicomyces hydei*	GZCC 23-0727^T^	PP512977	N/A	PP526731	PP526737
*Neohelicomyces lignicola*	CGMCC 3.25551^T^	PP639456	PP626600	PP596357	PP596483
*Neohelicomyces longisetosus*	NCYU-106H1-1-1^T^	N/A	MT939303	N/A	N/A
*Neohelicomyces macrosporus*	CGMCC 3.25552^T^	PP639457	PP626601	PP596358	PP596484
*Neohelicomyces maolanensis*	GZCC 23-0079^T^	PQ098529	N/A	PQ490683	PQ490677
*Neohelicomyces melaleucae*	CPC 38042^T^	MN567661	MN562154	MN556835	N/A
*Neohelicomyces pallidus*	CBS 271.52	AY856887	AY916461	N/A	N/A
*Neohelicomyces pallidus*	CBS 962.69	AY856886	AY916460	N/A	N/A
*Neohelicomyces pandanicola*	KUMCC 16-0143^T^	MH260307	MH275073	MH412779	N/A
*Neohelicomyces qixingyaensis*	CGMCC 3.25569^T^	PP639458	PP626602	PP596359	PP596485
*Neohelicomyces sexualis*	HGUP 24-0021^T^	PQ570861	PQ570844	N/A	N/A
*Neohelicomyces* sp.	GMBCC 2225	PX308848	PX308843	PX314510	PX314514
*Neohelicomyces* sp.	GMBCC 2217	PX308846	PQ737369	PX314508	PX314512
*Neohelicomyces submersus*	MFLUCC 16-1106^T^	KY320547	KY320530	N/A	MH551068
*Neohelicomyces subtropicus*	GZCC 23-0076^T^	PQ098530	PQ098492	PQ490685	PQ490679
***Neohelicomyces terrestris***	**GZCC 23-0399^T^**	**PX575662**	**PX575639**	**PX512845**	**PX512836**
***Neohelicomyces terrestris***	**GZCC 25-0660**	**PX575663**	**PX575640**	**PX512846**	**PX512837**
*Neohelicomyces thailandicus*	MFLUCC 11-0005^T^	MN913696	NR_171882	N/A	N/A
***Neohelicomyces tropicus***	**GZCC 25-0661^T^**	**PX575664**	**PX575641**	**PX512847**	**PX512838**
***Neohelicomyces tropicus***	**GZCC 25-0662**	**PX575665**	**PX575642**	**PX512848**	**PX512839**
*Neohelicomyces wuzhishanensis*	GZCC 23-0410^T^	PQ098532	PQ098494	PV768325	PV768334
*Neohelicomyces xiayadongensis*	CGMCC 3.25568^T^	PP639460	PP626604	PP596361	PP596487
*Neohelicomyces yunnanensis*	GZCC 23-0735^T^	PP664113	PP664109	N/A	N/A
*Tubeufia guttulata*	GZCC 23-0404^T^	OR030834	OR030841	OR046678	OR046684
*Tubeufia hainanensis*	GZCC 22-2015^T^	OR030835	OR030842	OR046679	OR046685
*Tubeufia javanica*	MFLUCC 12-0545^T^	KJ880036	KJ880034	KJ880037	N/A
*Tubeufia krabiensis*	MFLUCC 16-0228^T^	MH558917	MH558792	MH550985	MH551118
*Tubeufia latispora*	MFLUCC 16-0027^T^	KY092412	KY092417	KY117033	MH551119
*Tubeufia laxispora*	MFLUCC 16-0232^T^	KY092408	KY092413	KY117029	MF535287
*Tubeufia mackenziei*	MFLUCC 16-0222^T^	KY092410	KY092415	KY117031	MF535288
*Tubeufia muriformis*	GZCC 22-2039^T^	OR030836	OR030843	OR046680	OR046686
*Tubeufia nigroseptum*	CGMCC 3.20430^T^	MZ853187	MZ092716	OM022002	OM022001
*Tubeufia pandanicola*	MFLUCC 16-0321^T^	MH260325	MH275091	N/A	N/A

Note: “^T^” indicates ex-type strains. Newly generated sequences are in bold. “N/A” indicates unavailable data in GenBank.

The maximum likelihood (ML) analysis was carried out using RAxML-HPC v.8 on XSEDE (8.2.12) with a GTRGAMMA approximation and rapid bootstrap analysis followed by 1,000 bootstrap replicates (Stamatakis 2014). The substitution model was automatically tested by the server. Bayesian Inference (BI) analysis was performed using MrBayes on XSEDE (3.2.7a) via CIPRES (Stamatakis 2014). The aligned FASTA file was converted to a Nexus format file using AliView (Daniel et al. 2010). The best-fit evolutionary model for the individual datasets was determined using MrModeltest v.2.3.10 (Nylander et al. 2008). The GTR+G+I substitution model was selected for LSU, ITS, and *tef*1-*α*, whereas the SYM+I+G model was applied to *rpb2*. The posterior probabilities (BYPP) were determined based on Bayesian Markov chain Monte Carlo (BMCMC) sampling (Huelsenbeck and Ronquist 2001). Four simultaneous Markov chains were run for 10,000,000 generations, and trees were sampled every 1,000^th^ generation. The burn-in phase was set at 25%, and the remaining trees were used for calculating posterior probabilities (BYPP).

Phylogenetic trees were visualized using FigTree v.1.4.4 and edited with Adobe Illustrator CC 2019 (v.23.1.0; Adobe Systems, USA).

## ﻿Phylogenetic results

The phylogenetic positions of the four novel strains were assessed using a multi-locus phylogenetic approach. The concatenated sequence matrix comprised 3,431 characters (LSU: 1–856, ITS: 857–1,450, *tef1-α*: 1,451–2,362, and *rpb2*: 2,363–3,431) across 51 taxa. Base frequencies and rates were A = 0.249274, C = 0.247026, G = 0.255949, and T = 0.247750; substitution rates were AC = 1.139215, AG = 5.117176, AT = 2.501758, CG = 1.050796, CT = 8.734543, and GT = 1.000000. The distribution shape parameter α equaled 0.181304.

**Figure 1.**
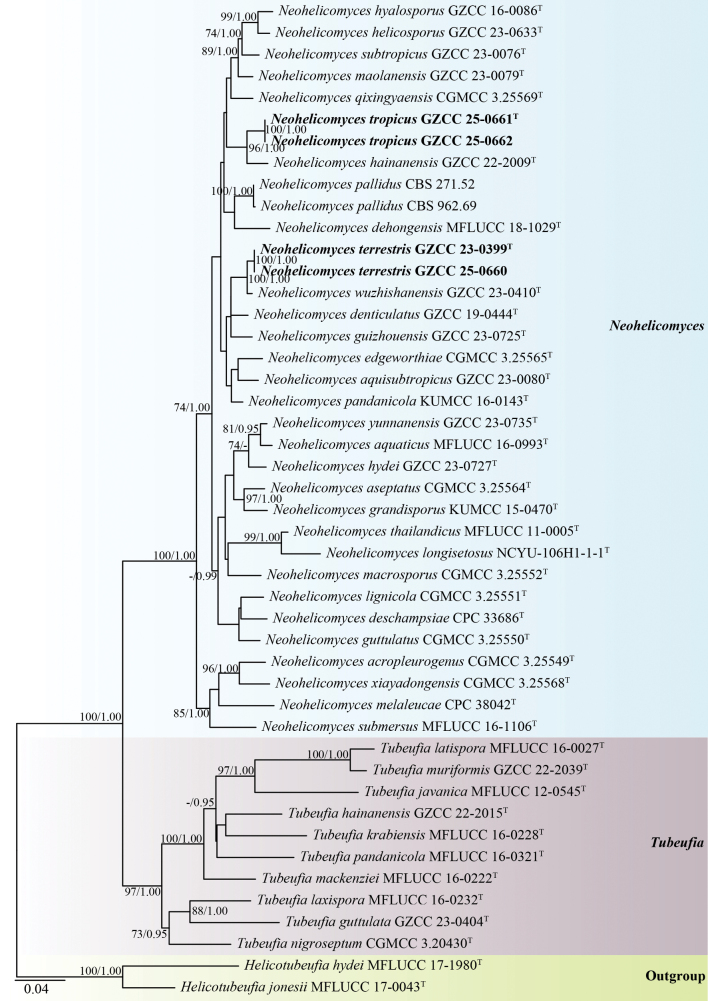
Phylogenetic tree generated from maximum likelihood (ML) analysis based on the combined LSU, ITS, *tef1-α*, and *rpb2* sequence data. Bootstrap support values for ML (≥ 70%) and BYPP (≥ 0.95) are indicated near their respective nodes. Both Maximum Likelihood (ML) and Bayesian Inference (BYPP) analyses produced congruent topologies. A hyphen (“-”) indicates a value lower than 70% for ML and a posterior probability lower than 0.95 for Bayesian inference. The tree is rooted with *Helicotubeufia hydei* (MFLUCC 17-1980) and *H. jonesii* (MFLUCC 17-0043). Ex-type strains are denoted with “^T,^” and newly obtained strains are in bold black fonts.

Based on the multi-gene phylogenetic tree (Fig. 1), our collections are identified as two distinct *Neohelicomyces* species within the family Tubeufiaceae (Tubeufiales, Dothideomycetes). The isolates GZCC 23-0399 and GZCC 25-0660 formed a sister clade to *N. wuzhishanensis* (GZCC 23-0410), with robust support of 87% ML and 1.00 BYPP. Furthermore, isolates GZCC 25-0661 and GZCC 25-0662 clustered together and formed a sister clade to *N. hainanensis* (GZCC 22-2009), with 95% ML and 1.00 BYPP support (Fig. 1).

## ﻿Taxonomy

### ﻿ Neohelicomyces terrestris

Taxon classificationFungiTubeufialesTubeufiaceae

T.H. Tan & J. Ma sp. nov.

609D7686-4B51-5D7B-9419-66681785D632

904429

Fig. 2

#### Etymology.

The species epithet “*terrestris*’’ refers to the terrestrial habitat of this fungus.

**Figure 2.**
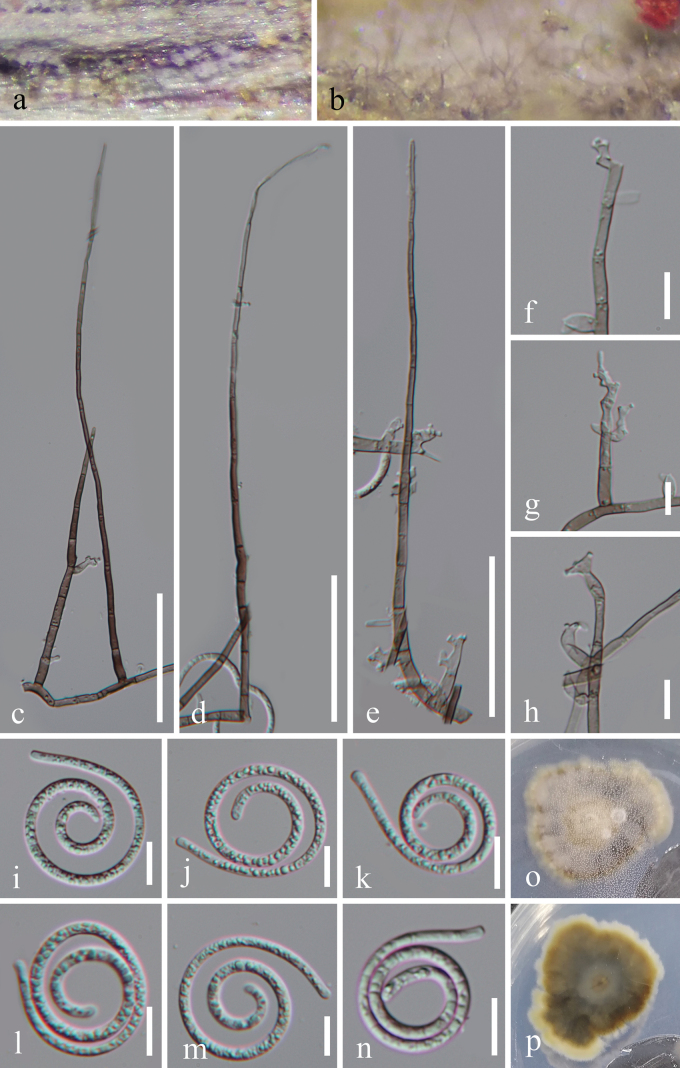
*Neohelicomyces terrestris* (HKAS 128950, holotype). a, b. Colonies on the host surface; c–e. Conidiophores and conidiogenous cells; f–h. Conidiogenous cells; i–n. Conidia; o, p. Colonies on PDA from above and below. Scale bars: 50 μm (c–e); 10 μm (f–n).

#### Holotype.

HKAS 128950.

#### Description.

***Saprobic*** on decaying wood in a terrestrial habitat. Sexual morph Undetermined. Asexual morph Hyphomycetous, helicosporous. ***Colo­nies*** on natural substrate superficial, effuse, gregarious, with masses of crowded, glistening conidia, white to brown. ***Mycelium*** partly immersed, partly superficial, composed of hyaline to pale brown, branched, septate, smooth hyphae. ***Conidiophores*** 106–212 × 3–4.5 μm (x̄ = 176 × 4 μm, n = 25), macronematous, mononematous, erect, cylindrical, flexuous, widest at the base, tapering towards narrow apex, branched or unbranched, septate, brown at base, subhyaline towards apex, thick-walled. ***Conidiogenous cells*** 7.5–25 × 2–4 μm (x̄ = 13.5 × 3.5 μm, n = 25), holoblastic, monoblastic, or polyblastic, integrated, terminal or intercalary, cylindrical, with denticles, subhyaline to pale brown, smooth-walled. ***Conidia*** solitary, acropleurogenous, helicoid, developing on tooth-like protrusion, 15–21 μm diam. and conidial filament 2.5–3.5 μm wide (x̄ = 17.5 × 3 μm, n = 20), 107–143 μm long (x̄ = 128.5 μm, n = 30), loosely coiled 2^1^/_2_–3 times, becoming loosely coiled in water, septate, guttulate, hyaline, smooth-walled.

#### Culture characteristics.

Conidia germinated on PDA and produced germ tubes within 11 h. Colonies on PDA reached 27 mm in diameter after 40 days of incubation at 25 °C, with an irregular shape, raised surface, and undulate margin, pale brown to brown; the reverse was brown to dark brown.

#### Material examined.

China • Hainan Province, Wuzhishan City, Shuimanhe tropical rainforest scenic area in Wuzhishan, 18°92′N, 109°63′E, on decaying wood in a terrestrial habitat, 4 November 2024, Ting-Hong Tan & Jian Ma, WZ66 (HKAS 128950, holotype), ex-type living cultures GZCC 23-0399 • *ibid*., WZ67 (GZAAS 23-0403, paratype), living culture GZCC 25-0660.

#### Notes.

Morphologically, *Neohelicomyces terrestris* (HKAS 128950) resembles *Parahelicomyces laxisporus* (HKAS 128943) in having macronematous, mononematous, erect, widest-at-the-base, tapering-towards-a-narrow-apex, brown-at-base, subhyaline-towards-apex, flexuous conidiophores; holoblastic, monoblastic or polyblastic, integrated, cylindrical conidiogenous cells with denticles, subhyaline to pale brown; and acropleurogenous, helicoid, aseptate, guttulate, hyaline conidia (Ma et al. 2024b). However, *N. terrestris* (HKAS 128950) differs from *Pa. laxisporus* (HKAS 128943) by its wider conidial diameter (up to 21 μm vs. 14.5–16 μm) and longer conidia (up to 143 μm vs. 80.5–124 μm) (Ma et al. 2024b). Phylogenetically, our isolates (GZCC 23-0399 and GZCC 25-0660) form a sister clade to *N. wuzhishanensis* (GZCC 23-0410), with 87% ML and 1.00 BYPP support (Fig. 1). Comparison of the LSU, ITS, *tef1-α*, and *rpb2* sequence data between *Neohelicomyces terrestris* (GZCC 23-0399) and *N. wuzhishanensis* (GZCC 23-0410) revealed nucleotide base differences of 5/486 bp (1%, including three gaps), 26/484 bp (5.4%, including 10 gaps), 25/937 bp (2.7%, including seven gaps), and 33/926 bp (3.6%, with no gaps), respectively. Therefore, based on both multi-gene phylogenetic analyses and morphological differences, we introduce *Neohelicomyces terrestris* as a novel species.

### ﻿ Neohelicomyces tropicus

Taxon classificationFungiTubeufialesTubeufiaceae

T.H. Tan & J. Ma sp. nov.

AEE65D81-C4A0-57F3-A5FA-E2DD51230827

904430

Fig. 3

#### Etymology.

The species epithet “*tropicus*” refers to the tropical climate in which the species occurs.

**Figure 3.**
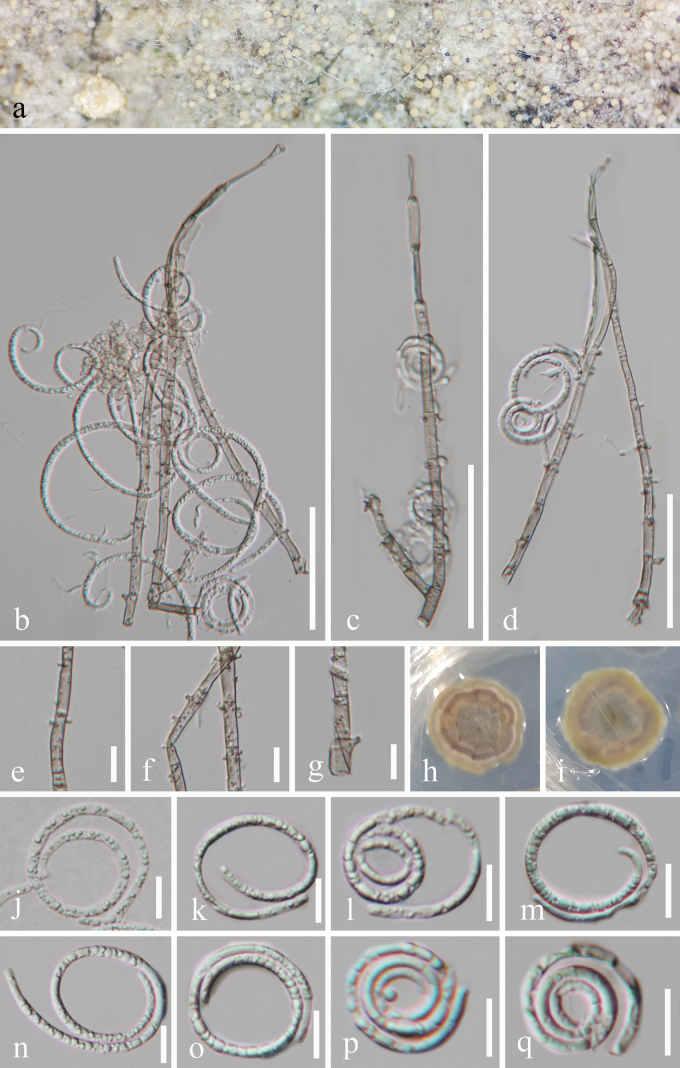
*Neohelicomyces tropicus* (GZAAS 25-0675, holotype). a. Colonies on the host surface; b–d. Conidiophores, conidiogenous cells, and conidia; e–g. Conidiogenous cells; **j**: Germinated conidium; k–q. Conidia; h, i. Colonies on PDA from above and below. Scale bars: 50 μm (b–d); 10 μm (e–g, j–q).

#### Holotype.

GZAAS 25-0675.

#### Description.

***Saprobic*** on decaying wood in a terrestrial habitat. Sexual morph Undetermined. Asexual morph Hyphomycetous, helicosporous. ***Colonies*** on natural substrate superficial, gregarious, with a little of crowded, glistening conidia, white. ***Mycelium*** partly immersed, partly superficial, composed of hyaline to pale brown, branched, septate, smooth hyphae. ***Conidiophores*** 139–175 × 4–5.5 μm (x̄ = 154 × 4.5 μm, n = 25), macronematous, mononematous, erect, cylindrical, straight or slightly flexuous, branched or unbranched, septate, subhyaline to pale brown, thick-walled. ***Conidiogenous cells*** 10–17 × 3–4 μm (x̄ = 14 × 3.5 μm, n = 25), holoblastic, monoblastic or polyblastic, integrated, terminal or intercalary, cylindrical, with denticles, subhyaline to pale brown, smooth-walled. ***Conidia*** solitary, acropleurogenous, helicoid, tapering towards the rounded ends, developing on tooth-like protrusion, 15.5–20 μm diam. and conidial filament 2–3.5 μm wide (x̄ = 17.5 × 2.5 μm, n = 20), 95–140 μm long (x̄ = 116 μm, n = 20), tightly coiled up to 3 times, becoming loosely coiled when the conidia are young and not becoming loose when mature in water, aseptate, guttulate, hyaline, smooth-walled.

#### Culture characteristics.

Conidia germinated on PDA and produced germ tubes within 14 h. Colonies on PDA reached 25 mm in diameter after 38 days of incubation at 25 °C with a circular shape, flat surface, and entire margin, pale brown to brown; the reverse was pale brown to brown.

#### Material examined.

China • Hainan Province, Baoting Li and Miao Autonomous County, Qixianling Hot Spring National Forest Park, on decaying wood in a terrestrial habitat, 2 November 2024, Ting-Hong Tan & Jian Ma, Q45 (GZAAS 25-0675, holotype), ex-type living culture GZCC 25-0661 • *ibid*., Q47 (GZAAS 25-0690, paratype), living culture GZCC 25-0662.

#### Notes.

In the phylogenetic analyses (Fig. 1), our isolates (GZCC 25-0661 and GZCC 25-0662) formed a well-supported sister clade to *Neohelicomyces hainanensis* (GZCC 22-2009), with 95% ML and 1.00 BYPP values. Morphologically, *N. tropicus* (GZAAS 25-0675) and *N. hainanensis* (GZAAS 22-2009) are nearly identical in their conidiophores, conidiogenous cells, and conidia (Lu et al. 2022). However, nucleotide comparisons among the LSU, ITS, *tef1-α*, and *rpb2* sequence data between *Neohelicomyces tropicus* (GZCC 25-0661) and *N. hainanensis* (GZCC 22-2009) revealed differences of 4/815 bp (0.5%, with no gaps), 23/482 bp (4.8%, including 18 gaps), 15/912 bp (1.6%, with no gaps), and 27/1,045 bp (2.6%, with no gaps), respectively. Therefore, based on the molecular data, we introduce *Neohelicomyces tropicus* as a novel species.

## ﻿Discussion

Helicosporous hyphomycetes share similar conidial characteristics, making accurate identification based solely on morphology challenging (Kuo and Goh 2018a, 2018b, 2021; Xiao et al. 2023; Ma et al. 2023, 2024b, 2025). Additional molecular evidence, particularly multi-gene phylogenetic analyses, **is** essential for accurate taxonomic delineation. However, accurate recognition of all key DNA markers used to identify and circumscribe helicosporous hyphomycetes depends on the availability and completeness of sequence data. The absence or partial representation of certain gene regions may lead to species misidentification. For instance, our newly isolated species, *Neohelicomyces tropicus* and *N. hainanensis*, exhibit only minor morphological differences and form sister clades in the phylogenetic tree (Fig. 1). When only ITS and LSU sequences are compared, the two taxa appear to represent the same species; however, further analyses of *tef1-α* and *rpb2* sequences, together with interspecific variation, support the recognition of *N. tropicus* as a distinct species. Similarly, some species of *Helicosporium*, *Neohelicosporium*, and *Tubeufia* require *tef1-α* and *rpb2* sequence data for accurate identification (Lu et al. 2018b; Xiao et al. 2023; Ma et al. 2024b).

Some helicosporous taxa exhibit similar conidial, conidiophore, and conidiogenous cell morphologies; however, multi-gene phylogenetic analyses have revealed that they belong to different genera (Lu et al. 2018b; Ma et al. 2024b). For instance, our newly introduced *Neohelicomyces terrestris* and certain *Parahelicomyces* species possess macronematous, mononematous, erect conidiophores that are widest at the base, tapering toward a narrow apex, brown at the base, subhyaline toward the apex, and flexuous. They also feature holoblastic, monoblastic to polyblastic, integrated, cylindrical conidiogenous cells with denticles that are subhyaline to pale brown and acropleurogenous, helicoid, aseptate, guttulate, hyaline conidia (Lu et al. 2018b; Ma et al. 2024b). Nevertheless, DNA sequence data clearly indicate that *N. terrestris* belongs to *Neohelicomyces* rather than *Parahelicomyces*. Furthermore, previous studies have demonstrated that certain morphologically similar helicosporous taxa are distributed across different genera, families, orders, and even classes (Lu et al. 2018b; Dayarathne et al. 2019; Jayasiri et al. 2019; Ma et al. 2024b). Therefore, accurate taxonomic identification of this group requires both morphological and molecular evidence.

Ecological factors appear to play a non-negligible role in the classification and diversification of helicosporous hyphomycetes (Ma et al. 2024b). These fungi are commonly associated with specific substrates such as decaying wood, submerged plant debris, or bamboo, which may influence their morphological adaptations and sporulation patterns. Therefore, ecological specialization may contribute not only to morphological differentiation but also to phylogenetic divergence within the group. Integrating ecological data with molecular evidence may thus provide a more comprehensive framework for understanding species boundaries and evolutionary relationships among helicosporous hyphomycetes.

## Supplementary Material

XML Treatment for Neohelicomyces terrestris

XML Treatment for Neohelicomyces tropicus

## ﻿Additional information

### Use of AI

No use of AI was reported.

### Author ORCIDs

Fan Gao https://orcid.org/0009-0003-9887-9729

Ting-Hong Tan https://orcid.org/0000-0002-2933-2696

Song Bai https://orcid.org/0000-0002-1972-2834

Chun-Fang Wu https://orcid.org/0009-0002-5166-0060

Ning-Ning Zhao https://orcid.org/0009-0004-4407-755X

Na Qiu https://orcid.org/0009-0002-1637-4720

Min Zhou https://orcid.org/0009-0009-6871-8928

Jian Ma https://orcid.org/0009-0008-1291-640X

### Data availability

All of the data that support the findings of this study are available in the main text.
